# Efficacy and safety of intra-arterial chemotherapy combined with intravesical chemotherapy for high-risk non-muscle invasive bladder cancer: A protocol for a systematic review and meta-analysis

**DOI:** 10.1097/MD.0000000000018516

**Published:** 2019-12-20

**Authors:** Xing Li, Haohao Ma, Kunpeng Shu, Lingdian Wang, Degang Ding

**Affiliations:** a Department of Urology; b Department of Orthopedics, Henan Provincial People's Hospital, People's Hospital of Zhengzhou University; c Department of Urology, Henan Provincial People's Hospital, People's Hospital of Henan University, People's Hospital of Zhengzhou University, Zhengzhou, Henan, China.

**Keywords:** efficacy, intra-arterial chemotherapy, intravesical chemotherapy, non-muscle invasive bladder cancer, safety

## Abstract

**Background:**

Non-muscle invasive bladder cancer (NMIBC) is the most common bladder cancer. Many studies have reported that intra-arterial chemotherapy (IAC) combined with intravesical chemotherapy (IVC) could effectively reduce the recurrence rate of NMIBC. The purpose of this study is to assess the efficacy and safety of IAC combined with IVC for patients with high-risk NMIBC.

**Methods:**

PubMed, Cochrane Library, Medline, Embase, Web of Science, and 4 Chinese databases will be searched for eligible studies published without language restrictions from their inception up August 31, 2019. Subgroup analysis will be mainly explored in study design, types of chemotherapy drugs, and sample size. Cochrane Collaboration Risk of bias Tool will be applied in evaluating the quality of enrolled articles. Statistical analysis will be carried out by the Stata version 14.0 software.

**Results:**

The primary outcome is recurrence-free survival (RFS). The secondary outcomes include overall survival (OS), progression-free survival (PFS), adverse reactions and toxicity grade coded by common toxicity criteria for adverse events.

**Conclusion:**

The findings of this study will provide latest evidence to verify whether IAC combined with IVC is more effective and safer than IVC alone for patients with high-risk NMIBC.

**PROSPERO registration number:**

CRD42019146847

## 1 Introduction

As one of the most common malignant tumors of urinary system, bladder cancer (BC) has become a major health issue worldwide.^[1]^ In terms of its updated incidence, approximately 61,700 men and 18,770 women will be newly diagnosed with BC in the United States in 2019 alone.^[2]^ Non-muscle invasive bladder cancer (NMIBC) refers to the limited aggression of cancer tissue to the mucosa or submucosa of the bladder wall (stage Ta, Tis, T1), with the incidence about 75% of the total.^[3]^ Patients with NMIBC should be treated with standard therapy that is transurethral resection of bladder tumor (TURBT) combined with intravesical instillation, based on risk stratification.^[4]^ Although there are many types of drugs for intravesical instillations, including Bacillus Calmette-Guerin (BCG), epirubicin, pirarubicin, and gemcitabine, etc, NMIBC still has a high recurrence rate, especially for high-risk patients.^[5]^ Moreover, in China, BCG has not been approved by the Food and Drug Administration of the People's Republic of China until late 2015 and is still not commonly applied in most of Primary Medical institutions due to lack of supply and high cost. Thus, the exploring of novel therapeutic drugs and strategies for patients with NMIBC is urgent and necessary at present.

Intra-arterial chemotherapy (IAC), initially introduced by Japanese investigator Kubota et al^[6]^ in 1986, has been gradually applied in the treatment of tumors because of its less side effects of chemotherapy and better anticancer efficacy. The idea behind of IAC is to infuse chemotherapeutic drugs into cancer patients through the main blood supply arteries of tumors, which not only can increase the local blood concentration of tumor tissue, but also reduces the systemic toxicity induced by chemotherapeutic drugs.^[7]^ Previous studies have shown that IAC is more effective and safer than intravenous chemotherapy for treating certain malignant tumors, such as retinoblastoma, pancreatic cancer, and gallbladder cancer.^[8–10]^ In the treatment of BC, IAC was primarily used as a palliative therapeutic schedule for patients with muscle invasive bladder cancer (MIBC) who were unable to undergo radical cystectomy or had a strong desire to retain bladder function.^[11,12]^

Recently, the efficacy of IAC combined with intravesical chemotherapy (ICV) was gradually demonstrated in preventing recurrence and progression of NMIBC after TURBT, especially for high-risk patients who may need cystectomy.^[13–16]^ Although the existing research on IAC for NMIBC patients is limited and the clinical experience of this therapy is still insufficient so far, interest and attention on this novel pathway of chemotherapy has been increased and strengthened. Therefore, we plan to perform a systematic review and meta-analysis on the published trials to comprehensively assess the efficacy and safety of IAC combined with IVC in patients with NMIBC.

## 2 Method

### 2.1 Study registration

We have registered this study protocol on the PROSPERO and got a unique registration number (CRD42019146847). The systematic review protocol will be strictly performed following the Cochrane Handbook for Systematic Reviews and the guidelines of Meta-Analyses protocols (PRISMA-P).

### 2.2 Ethics

Since all analyses will be based on previously published articles, ethical approval, and patient consent is not needed.

### 2.3 Inclusion criteria for study selection

#### 2.3.1 Types of studies

Randomized controlled trials (RCTs), Hi-Q (high quality) prospective cohort trials, and case–control studies will be considered that should be completed and compare IAC combined with IVC versus IVC alone in high-risk NMIBC patients after transurethral resection (including laser resection).

#### 2.3.2 Types of participants

Patients who were diagnosed with high-risk NMIBC and underwent transurethral resection will be included, regardless of age, sex, race, occupation, education, etc. However, patients with other types of malignancies at the same time will be excluded.

#### 2.3.3 Types of interventions

All patients in the experimental group receive intra-arterial chemotherapy combined with intravesical chemotherapy after transurethral resection. For patients in the control group, intravesical chemotherapy is the only treatment.

#### 2.3.4 Types of outcome measurements

Primary outcomes: The recurrence-free survival (RFS) of patients will be analyzed as the primary outcome.

Secondary outcomes: The secondary outcomes include overall survival (OS), progression-free survival (PFS), adverse reactions (including nausea/vomiting, hypo-leukemia, neutropenia, fever, liver dysfunction, renal dysfunction, etc), and toxicity grade coded by common toxicity criteria for adverse events.

### 2.4 Search methods for identification of studies

#### 2.4.1 Electronic searches

We will perform a systemically retrieval in relevant electronic databases which include^[5]^ English databases (PubMed, Cochrane Library, Medline, Embase, Web of Science) and^[4]^ Chinese databases (Chinese Biomedical Literature Database [CBM], China National Knowledge Infrastructure [CNKI], Wanfang China database, and Chinese Scientific Journal Database [VIP]). The initial retrieval process will be carried out by 2 reviewers (XL and KS) independently up to August 31, 2019.

#### 2.4.2 Searching strategy

The search strategy consisting of MeSH terms and related synonym for PubMed is listed in Table 1. Appropriate modification will be made to the search strategy as required for other electronic databases.

**Table 1:**
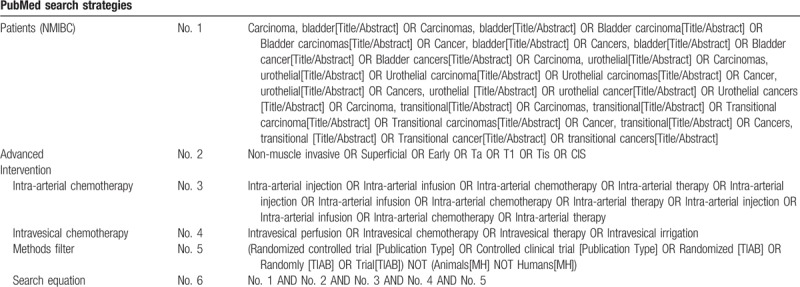
PubMed search strategies.

### 2.5 Data collection and analysis

#### 2.5.1 Date collection

**Figure 1:**
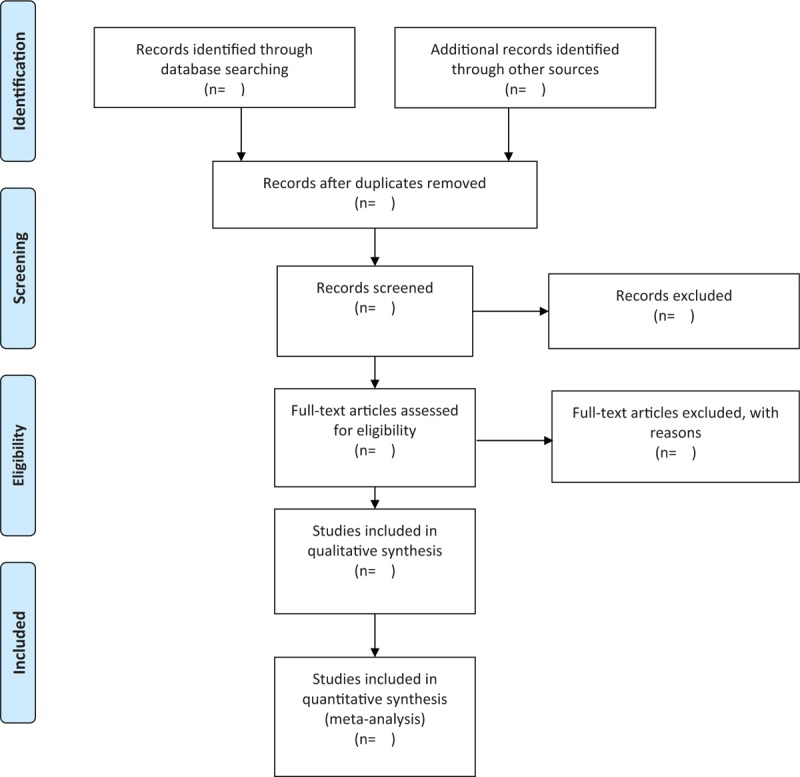
PubMed search strategies.

The flow diagram of literature search and selection is shown in Fig. 1. Two authors will extract the data from original publications independently, and any disagreements will be solved through discussion with a third reviewer if necessary.

Publication information is as follows:

- Study characteristics and methodology: name of first author, publication date, region, sample size, randomization, study design, follow-up duration, and withdrawals, etc.
- Participant characteristics: age, sex, tumor grade, pathological T stage, tumor size, tumor number, and inclusion criteria, etc.
- Interventions: means of chemotherapy, treatment cycle, types of chemotherapy drugs, dosage, and duration of administration, etc.
- Outcome and other data: recurrence-free survival, overall survival, progression-free survival, recurrence-free rate, 95% confidence intervals (CIs), adverse reactions, and toxicity grade, etc.

#### 2.5.2 Assessment of risk of bias

Two reviewers will evaluate the potential risk of bias and quality of each included study in strict based on the Cochrane Handbook for Systematic Reviews of Interventions. The assessment items are as follows: random sequence generation; allocation concealment; blinding method for participants and researchers; blinding of outcome assessment; incomplete result data addressed; selective outcome reporting, and other bias.^[17]^ Each item will be evaluated as low, high, or an unclear risk of bias.

#### 2.5.3 Data analysis

The available data will be synthetically analyzed using the Stata version 14.0 software (Stata Corporation, College Station, TX). The heterogeneities of included studies will be assessed by applying chi-square test and *I*^2^ statistic. A random-effects model will be constructed if the heterogeneity has statistical significance (*P* < .05 or *I*^2^ > 50%); otherwise, the fixed-effect model will be adopted. Meanwhile, subgroup analysis will be conducted to explore the sources of any heterogeneity. For dichotomous variable, the data will be expressed as relative risk (RR) and 95% CIs. For continuous variable, mean difference (MD) or standard MD (SMD) with 95% CIs of the data will be calculated for reporting the final result.

#### 2.5.4 Subgroup analysis

We will carry out subgroup analysis if there is significant heterogeneity (*I*^2^ > 50%) and the available data are sufficient. Subgroup analysis will be mainly explored in study design, types of chemotherapy drugs, and sample size.

#### 2.5.5 Sensitivity analysis

Where appropriate, we will operate a sensitivity analysis to test the stability and robust of the statistical result by excluding each single study.

### 2.6 Publication bias

Funnel plots and Egger test will be conducted to check publication bias if the quantity of trials included in the final study is ≥10. If reporting bias is suspected in a trial, we will consult the study author to acquire more information at an appropriate time.

## 3 Discussion

Non-muscle invasive bladder cancer (NMIBC) accounts for about 3 quarters of all cases of BC, and high-risk NMIBC is more likely to recur or progress to MIBC. Although the standard treatment of TURBT and adjuvant intravesical chemotherapy has been widely promoted, the prognosis of patients with high-risk NMIBC is still unsatisfactory. Therefore, novel therapies that could effectively reduce the recurrence rate and alleviate the side effects of chemotherapy are what we need to explore urgently now. Intra-arterial chemotherapy combined with intravesical chemotherapy (IAC+IVC) is a new method for treating high-risk NMIBC, and several clinical studies have confirmed that IAC combined with IVC can benefit patients more than traditional IVC alone. The aim of this study is to further evaluate the efficacy and safety of IAC combined with IVC for high-risk NMIBC comprehensively and provides reliable evidence for urologists to establish individualized and precise therapeutic regimens for patients.

## Author contributions

**Conceptualization**: Xing Li, Degang Ding.

**Data curation**: Xing Li, Haohao Ma, Kunpeng Shu, Lingdian Wang.

**Formal analysis**: Xing Li, Haohao Ma.

**Investigation**: Xing Li, Haohao Ma, Kunpeng Shu, Lingdian Wang, Degang Ding.

**Methodology**: Xing Li, Haohao Ma, Kunpeng Shu, Lingdian Wang, Degang Ding.

**Project administration**: Xing Li, Haohao Ma.

**Resources**: Degang Ding.

**Software**: Xing Li, Haohao Ma, Kunpeng Shu.

**Supervision**: Degang Ding, Lingdian Wang.

**Writing – original draft**: Xing Li, Haohao Ma, Kunpeng Shu, Lingdian Wang, Degang Ding.

**Writing – review & editing**: Xing Li, Haohao Ma, Kunpeng Shu, Lingdian Wang, Degang Ding.
